# Effect of Mg doping on morphology, photocatalytic activity and related biological properties of Zn_1−x_Mg_x_O nanoparticles

**DOI:** 10.3906/kim-2004-9

**Published:** 2020-08-18

**Authors:** Bestenur YALÇIN, Doğan AKCAN, İbrahim Ertuğrul YALÇIN, Mehmet Can ALPHAN, Kenan ŞENTÜRK, İbrahim İlker ÖZYİĞİT, Lütfi ARDA

**Affiliations:** 1 Department of Medical Laboratory Techniques, Vocational School of Health Services, Bahçeşehir University İstanbul Turkey; 2 Department of Mathematics Engineering, Faculty of Engineering and Natural Sciences, Bahçeşehir University, İstanbul Turkey; 3 Department of Civil Engineering, Faculty of Engineering and Natural Sciences, Bahçeşehir University, İstanbul Turkey; 4 Department of Electric Electronic Engineering, Faculty of Engineering and Natural Sciences, Bahçeşehir University, İstanbul Turkey; 5 Department of Mechatronics Engineering, Faculty of Engineering and Architecture, İstanbul Gelişim University, İstanbul Turkey; 6 Department of Biology, Faculty of Science and Arts, Marmara University, İstanbul Turkey; 7 Department of Biology, Faculty of Science, Kyrgyz-Turkish Manas University, Bishkek Turkey; 8 Department of Mechatronics Engineering, Faculty of Engineering and Natural Sciences, Bahçeşehir University, İstanbul Turkey

**Keywords:** Sol-gel method, Mg doped ZnO, hemolytic activity, antibacterial properties, *E. coli*

## Abstract

The objective of this study is to synthesize ZnO and Mg doped ZnO (Zn_1−x_Mg_x_O) nanoparticles via the sol-gel method, and characterize their structures and to investigate their biological properties such as antibacterial activity and hemolytic potential.Nanoparticles (NPs) were synthesized by the sol-gel method using zinc acetate dihydrate (Zn(CH_3_COO)_2_.2H_2_O) and magnesium acetate tetrahydrate (Mg(CH_3_COO)_2_.4H_2_O) as precursors. Methanol and monoethanolamine were used as solvent and sol stabilizer, respectively. Structural and morphological characterizations of Zn_1−x_Mg_x_O nanoparticles were studied by using XRD and SEM-EDX, respectively. Photocatalytic activities of ZnO and selected Mg-doped ZnO (Zn_1−x_Mg_x_O) nanoparticles were investigated by degradation of methylene blue (MeB). Results indicated that Mg doping (both 10% and 30%) to the ZnO nanoparticles enhanced the photocatalytic activity and a little amount of Zn0.90 Mg0.10 O photocatalyst (1.0 mg/mL) degraded MeB with 99% efficiency after 24 h of irradiation under ambient visible light. Antibacterial activity of nanoparticles versus *Escherichia coli* ( *E. coli* ) was determined by the standard plate count method. Hemolytic activities of the NPs were studied by hemolysis tests using human erythrocytes. XRD data proved that the average particle size of nanoparticles was around 30 nm. Moreover, the XRD results indicatedthat the patterns of Mg doped ZnO nanoparticles related to ZnO hexagonal wurtzite structure had no secondary phase for x ≤ 0.2 concentration. For 0 ≤ x ≤ 0.02, NPs showed a concentration dependent antibacterial activity against *E. coli* . While Zn_0.90_Mg_0.10_ O totally inhibited the growth of *E. coli* , upper and lower dopant concentrations did not show antibacterial activity.

## 1. Introduction

Synthesis, characterization, and application of nanoscale materials lead to the design of new functional materials and devices with unique properties. Among nanosized materials, metal-oxide nanoparticles have an incredibly big potential in many research areas due to their small particle size, large surface area and advanced chemical, optical, morphological and antibacterial properties. Metal oxide NPs can be synthesized by using biological systems as bacteria, fungi and yeast [1–4] or synthetic pathways like hydrothermal method [5,6], sol-gel method [7–11] and chemical vapor deposition [12]. Metal-oxide NPs find applications in textile industry [13,14], electronics [15,16,17], gas sensing applications [18–20], in medical applications like selective destruction of tumor cells [21], intracellular drug delivery [22] and some diagnostic applications.

Among metal-oxide NPs, zinc oxide (ZnO) has a special biological and medical importance [23–26]. ZnO NPs are regarded as biosafe due to their low toxicity in some researches [27, 28] and they are also assigned as GRAS (generally recognized as safe) by FDA (US Food and Drug Administration) [29,30]. In a related study, the effects of ZnO nanowires (average diameter of 1 μm and the average length of 200 μm) on Hela cell line (a type of epithelial cell) and L-929 cell line (connective tissue cells) were investigated by measuring the activity of succinate dehydrogenase (SDH). ZnO nanowire concentrations of 0.1, 1, 10, and 100 μg/mL were studied, an it was stated that ZnO nanowires were completely biocompatible and biosafe at cellular level at concentrations lower than 100 μg/mL [23]. Moreover, zinc is one of the essential elements both in plants and animals [31–33]. It has an important role in enzyme activity and acts as a cofactor of some enzymes such as carbonic anhydrase carboxypeptidases, dipeptidases, DNA and RNA polymerases, pyruvate carboxylase and alkaline phosphatase [34,35].

Synthetic materials in contact with blood may cause damage to the walls of erythrocyte cells and may trigger various cell reactions, such as thrombus formation [36–38]. The hemolytic potential of a material is a measure of hemolysis that is caused by any material in contact with blood. ZnO NPs also show disruptive effect on biofilm formation and inhibit hemolysis so that they are assigned as hemocompatible [39]. Zhang et al. have shown that ZnO NP suspensions with 0.75 mg/ml (mg ZnO/ml physiological saline) concentration cause no apparent hemolysis [40]. Other studies on the healing of skin wounds showed that ZnO is highly effective in promoting wound healing by increasing cell reepithelization [41].ZnO NPs are currently used in pain easing and itch relieving commercial ointments [42–44].

The antibacterial activities of metal-oxide nanoparticles are noteworthy as a new technique that can replace the traditional methods in which organic antibiotics are used. The most remarkable advantages of inorganic antimicrobial agents are being of safe and stable, as compared totheir organic equivalents.

Metal-oxide nanoparticles which contain essential mineral elements like Zn, Mg and Ca (ZnO, MgO and CaO) also show antimicrobial action which their equivalent micro and macro sized materials do not possess [45–47]. In some studies, it was shown that, ZnO NPs show selective toxicity against bacteria and act as an inert nanomaterial on human cells [48–51]. Several mechanisms were introduced to the literature about the antibacterial action of ZnO NPs. In some researches, release of metal ions like Zn^2+^ from NP structure was held responsible from the antibacterial activity [52]. However, the solubility of metal oxides like ZnO is very low and concentration dependent. Besides, some researches showed that as an essential mineral, low concentrations of Zn^2+^ ion (0.01–1.00 mM) may act as a nutrient and can enhance the growth of bacteria [53]. Another accepted mechanism of antibacterial action is the electrostatic interaction between cell walls and nanoparticles and the accumulation of nanoparticles on cell membrane [54]. According to Stoimenov, the total electrical charge of bacteria cells is negative at biological pH because of the presence of negatively charged carboxylate groups on cell wall. On the contrary, ZnO NPs are positively charged [54]. These opposite charges cause an attraction between bacteria cell and ZnO NPs which results in cell death. Pati et al. [55] have shown the disruptive effect of ZnO NPs on the integrity of bacteria cell wall and oxidative stress resistant genes in cells. ZnO NPs are known as stable at neutral or biological pH. On the contrary, in acidic media (pH 4.5), they dissolve to form Zn^2+^ ions [56]. ZnO particles accumulate on cell wall and introduce into the cell, dissolve in lysosomes having a pH range of 4.5–5.0 [57] and cause necrotic cell death [58,59]. Thus, although Zn is essential for the continuity of metabolic activities in the cell, the uncontrolled increase in Zn concentration as a result of the dissolution of ZnO nanoparticles in the cell is stated to be areason of cell death.

Besides the other mechanisms, the main responsible effect related to the antibacterial activity of nano sized ZnO and its derivatives causing intracellular death of bacteria is the light catalyzed formation of reactive oxygen species (ROS) such as hydroxyl radical (*OH), superoxide anion radical (O^-*^_2_) and hydrogen peroxide (H_2_O_2_) [53,60–62]. ZnO nanoparticles may cause light catalyzed formation of ROS like O^-*^_2_, * OH and H_2_O_2_. When the concentration of ROS exceeds the defense capacity of the cell, adverse biological consequences result [63].

In this study, the effects of Mg doping ratios on morphology, photocatalytic activity and biological effects of ZnO nanoparticles were investigated. ZnO and Mg-doped ZnO(Zn_1−x_Mg_x_O) nanoparticles were synthesized via the sol-gel method and examined for their antibacterial activity and hemolytic potential.The effects of high doping levels (30%) of Mg on Zn_1−x_Mg_x_O NPs were investigated and results were compared with pure ZnO nanoparticles.

## 2. Materials and methods

Zinc acetate dihydrate [Zn(CH_3_COO)_2_.2H_2_O, Merck & Co., Inc., Kenilworth, NJ, USA], and magnesium acetate tetrahydrate [Mg(CH_3_COO)_2_.4H_2_O, Alfa Aesar, Haverhill, MA, USA] were used as precursors without purification. HPLC grade methanol (Merck & Co., Inc.) and monoethanolamine (Merck & Co., Inc.) were used as solvent and sol stabilizer respectively. Tyriptic soy agar (TSA) and tyriptic soy broth (TSB) were purchased from Merck & Co., Inc. Distilled deionized water (DDW) was obtained from a Human Zeneer power 1 water purification system (Human Corporation, Seoul, Korea). Phosphate buffer solution (PBS, pH 7.35) for hemolysis tests was prepared using NaCl (8 g/L), KCl (0.2 g/L), Na_2_HPO_4_.2H_2_O (1.78 g/L) and KH_2_PO_4_ (0.24 g/L) andsterilized by autoclaving at 1 atm and 121 o C for 15 min. MeB (Methylene Blue - C_16_H_18_N_3_ClS.3H_2_O) was used as model organic dye in photocatalytic activity measurements.

### 2.1. Synthesis of ZnO and Zn_1−x_Mg_x_ O nanoparticles

**Figure 1:**
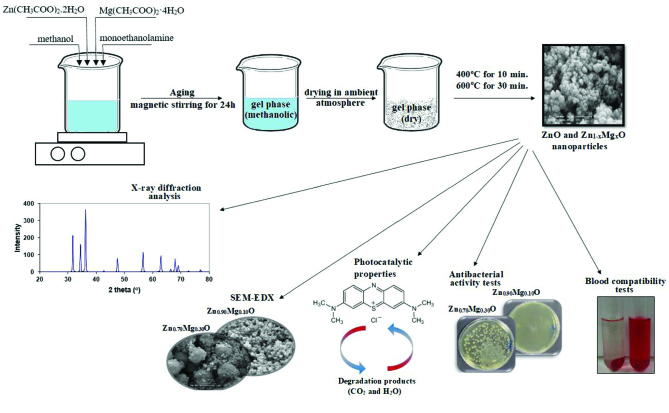
Flow chart for the synthesis and characterization of ZnO and Zn_1−x_Mg_x_O (x = 0.0, 0.01, 0.02, 0.04, 0.05, 0.10, 0.20, and 0.30) nanoparticles.

ZnO and Mg doped ZnO nanoparticles were synthesized by the sol-gel method. In a typicalsynthesis (Figure 1), methanolic precursor solutions (Zn(CH_3_COO)_2_.2H_2_O and Mg(CH_3_COO)_2_.4H_2_O) were prepared with a concentration of 0.25 mol/L. Precursor solutions then aged under magnetic stirring overnight.

To form the gel phase, aging solutions were evaporatedat room temperature under magnetic stirring. Then, heat treatment was applied at 400 °C for 10 min to combust the organic residues in the gel phase and at 600 °C for 30 min to obtain an accurate crystal orientation.

### 2.2. Structural analysis

Phase compositionsand crystal structures were characterized by the X-ray diffraction (XRD-Bruker D8 Advance) measurements. Surface morphologies were identified using scanning electron microscopy (SEM-FEI Quanta FEG 250). Energy dispersive X-ray spectroscopy and mapping analysis (SEM-EDX) was used to identify the elemental composition and quantitative concentrations of the constituting elements in nanoparticles.

Nanoparticle suspensions used in the antimicrobial activity tests and hemolytic activity experiments were prepared using DDW and PBS, respectively. Structural stability of suspended nanoparticles in aqueous medium was investigated by determination of Zn and Mg element concentrations in solution phase by the ICPOES (inductively coupled plasma optical emission spectroscopy – PerkinElmer-Optima 7000 DV, PerkinElme Corporation, Waltham, MA USA) analysis. Colloidal nanoparticle suspensions in distilled water and the PBS were centrifuged at 2000 rpm for 3 min prior to analysis and supernatant was used. Samples were read after calibrating the ICP-OES device with Zn and Mg standards.

### 2.3. Evaluation of photocatalytic properties

The photocatalytic performances of ZnO and Zn_1−x_Mg_x_O nanoparticles were investigated by measuring the photocatalytic degradation rate of MeB similar to the procedure given by Ali et al. [64]. Initial dye and nanoparticle concentrations were 1.0 ×10^−5^M and 1.0 mg/mL respectively. In a typical photocatalytic degradation experiment, selected samples from Mg doped ZnO nanoparticles and pure ZnO were dispersed in 100 mL of aqueous MeB solution and magnetic stirring was applied for 30 min in the dark to ensure adsorption/desorption equilibrium. Aqueous nanoparticle-MeB dispersions were then irradiated under ambient visible light while being continuously stirred. Also, characteristics of ambient light were examined using CCS200-compact spectrometer and a mixture of light in different wavelengths (436 nm, 546 nm, and 611 nm) were detected.

At appropriate time intervals, constant volume aliquots were taken and then centrifuged at 3000 rpm for 5 min to remove suspended ZnO or Zn1−x Mgx O nanoparticles. The instant concentrations of MeB were determined usinga UV/vis spectrophotometer (Shimadzu UV mini 1240, Shimadzu Corp., Kyoto, Japan) at a wavelength of 664 nm using DDW as reference.

The photocatalytic degradation of MeB followed the first order kinetics and corresponding rate constants were determined by the following equation [65];

 ln( A0/A)=kt

where A_o_ and A were the absorbances of MeB solution after adsorption equilibrium and after the time (t) respectively. k was the first order rate constant for the photocatalytic degradation. Moreover, the decolorization efficiency of MeB was estimated by the following equation [66];

Decolorization (%) =(C0-C/C0)x100

where C and C represent the initial concentration of MeB before irradiation, and the concentration of MeB after a certain irradiation time (t), respectively.

### 2.4. Antibacterial activity tests

Antibacterial activity of Zn_1−x_Mg_x_O NPs was determined by the standard agar plate count method using *E. coli* as model microorganism. Three different concentrations (0.5, 1.0, and 5.0 mg/mL) of ZnO and Zn_1−x_Mg_x_O nanoparticle suspensions were prepared in sterile test tubes by dispersing the NPs in DDW. Escherichia col K12 strain (Invitrogen, France) was used as a model organism in this study. Bacteria were cultivated in tyriptic soy broth containing 5 g/L of yeast extract, 10 g/L bactotryptone, and 10 g/L NaCl. After an overnight cultivation at 37 °C, optical density (OD) of bacterial suspension was determined at 600 nm. Then suspension was diluted using fresh tyriptic soy broth to final concentration of 1.0×105 cell forming unit(cfu)/mL. 150 μL of this bacterial suspension was inoculated onto each nanoparticle suspension and samples were incubated at 37 °C for 20 h. Test tubes were stirred at 200 rpm under magnetic stirring to keep the contact of NPs and bacteria during incubation period. At the end of incubation time, 50 μL from each test sample was spreaded out onto a TSA plate uniformly, then, plates were incubated at 37 °C for 20 h and the colonies were counted as a final step. Four replicates of each nanoparticle concentration were tested.

### 2.5. Blood compatibility tests

Human whole blood samples were used in the experiments and hemolytic activity of NPs were investigated according to earlier reports [67]. 4 mL of human blood anticoagulated with trisodium citrate solution (0.108 mM) was added to 10 mL of Ca and Mg free PBS solution and centrifuged at 2000 rpm for 5 min to separat the erythrocytes from plasma. After the supernatant was decanted, RBCs were diluted to 100 mL with PBS. 1.25 mL of this RBC stock solution was added to 5.0 mL of NPs suspensions in test tubes with different concentrations. RBCs suspended in DDW were used as positive control test (100% hemolysis), while RBCs solution dispersed in PBS (pH 7.4) was selected as negative control test (0% hemolysis). All dispersions were incubated under magnetic stirring at 200 rpm at 37 °C for 3 h and then centrifuged at 10,000 g for 3 min using a microcentrifuge. Three replicates of each sample were tested. Hemolysis ratios were calculated by measuring the absorbance value (ABS) of the supernatant solution at 545 nm using a UV-vis spectrophotometer.

## 3. Results

### 3.1. Structural analysis

**Figure 2:**
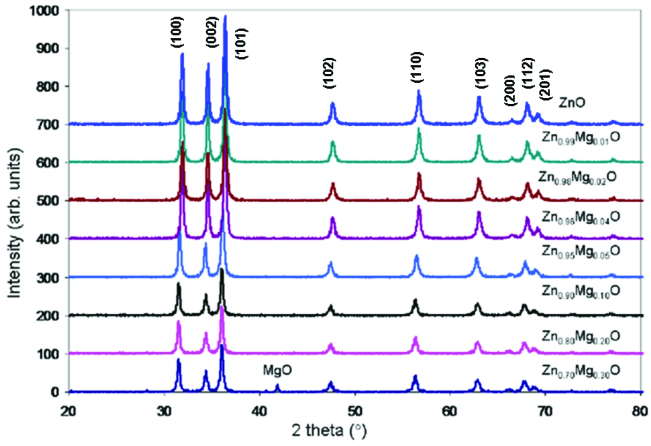
XRD patterns of ZnO and Zn_1−x_Mg_x_O nanoparticles.

Structural, electrical, magnetic and antibacterial properties of ZnO and ZnO doped with transtion metals strongly depend on the single phase of resulting materials with growth condition. The X-ray diffraction was used to analyze the phase and crystal structure of Zn_1−x_Mg_x_O (x = 0.0, 0.01, 0.02, 0.04, 0.05, 0.10, 0.20, and 0.30) samples synthesized by the sol-gel method which were annealed at 600 °C in air displayed in Figure 2. The XRD patterns of Mg doped ZnO nanoparticles showed the characteristic peaks of ZnO hexagonal wurtzite structure without secondary phases that for samples having x ≤ 0.20 concentration, whereas for the higher concentration values an additional MgO phase were observed. The peak (101) was found as the highest peak among all the other indexed peaks (Figure 2).

Zn_1−x_Mg_x_O (x = 0.01, 0.02, 0.04, 0.05, 0.10, 0.20, and 0.30) nanoparticle, concentration-dependent crystallite sizes (D), lattice parameters (a and c), volume of unit cell, dislocation density (δ) (amount of defect in sample), position and displacement of atoms (u) and bond length (L) were calculated and listed in Table by using XRD analyzes. The detailed evaluations of D, u, V, and L parameters were given below.

**Figure 3:**
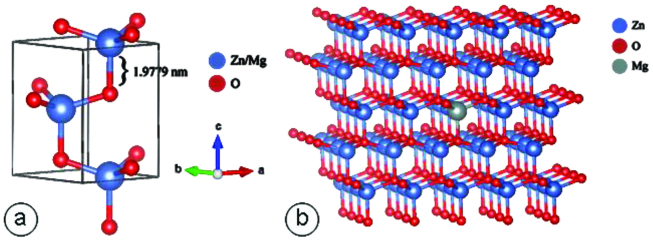
Illustrations of a. the unit cell, and b. the crystal structure of Zn_0.99_Mg_0.01_O nanoparticle.

The unit cells of Zn_0.99_Mg_0.01_O and the crystal structure of Zn_0.99_Mg_0.01_O were given in Figure 3. VESTA visualization package was used to illustrate the unit cell of hexagonal Mg doped ZnO as shown in Figure 3a, and randomly 1% Mg-doped to ZnO super cell (Figure 3b) by experimentally obtained parameters which were also displayed in Table.

The average crystallite size was calculated from the XRD peak width of (101) based on the Debye–Scherrer equation [7, 9],

D=Kλβhklcos(θ)

where β_hkl_ is the integral half width, K is a constant equal to 0.90, λ is the wavelength of the incident Xray (λ = 0.1540 nm), D is the crystallite size, and θ is the Bragg angle. The particle size calculated for synthesized ZnMgO nanoparticles was in the range of 21.6–26.88 nm.

**Table:** Concentration dependent lattice parameters, atomic packing factor (c/a), crystallite sizes (D), volume of the unit cell, dislocation density (δ) (the amount of defect in the sample), the locality of the atoms and their displacement (u), and bond length (L).

Samples	A(Å)	C(Å)	C/A	D (NM)	Volume V(Å^3^)	Dislocation density	U	Bond length (Å)	Microstrain
ZN_0.99_MG_0.01_O	3.249	5.210	1.604	26.88	47.629	0.00138	0.3796	1.9779	0.00129
ZN_0.98_MG_0.02_O	3.250	5.208	1.602	26.42	47.638	0.00143	0.3798	1.9780	0.00131
ZN_0.96_MG_0.04_O	3.250	5.204	1.601	24.2	47.610	0.00171	0.3800	1.9777	0.00143
ZN_0.95_MG_0.05_O	3.250	5.202	1.600	23.8	47.597	0.00177	0.3801	1.9775	0.00146
ZN_0.90_MG_0.10_O	3.251	5.195	1.598	22.9	47.532	0.00191	0.3805	1.9767	0.00151
ZN_0.80_MG_0.20_O	3.251	5.190	1.596	21.8	47.509	0.00210	0.3808	1.9764	0.00159
ZN_0.70_MG_0.30_O	3.252	5.182	1.593	21.6	47.468	0.00214	0.3813	1.9759	0.00160

The lattice constants a and cwere calculated with the following formula [7,9]:

1dhkl2=43(h2+hkk2a2)+l2c2

The volume of the unit cell of hexagonal system was calculated by the following equation:

V=0.866xa2xc

ZnO bond length was calculated by the following equation [7,9]:

L=(a23)+[0.5-u]2c2

where a and c are lattice constants of ZnO and u is the wurtzite structure which can be found as

u=(a23c2)+0.25

In a structure, both doping ratio and annealing temperature, are predominantly affecting strain. Therefore, microstrain (ε) was calculated by the following equation:

ε=(β1/2cosθ)/4

These parameters were presented in Table.

As can be seen from Table, the lattice parameter a and c of the Mg-doped ZnO nanoparticles are in the range of 3.249–3.252 Å and 5.182–5.21 Å, respectively. It was observed that when Mg concentration increased, the lattice parameter a and microstrain increased, and the lattice parameter c and cell volume decreased. There was a reduction in c/a lattice parameter ratio. This was expected since Mg ions replace Zn ions in the lattice, as the Mg (0.59 Å) ions have smaller ionic radii than Zn ions (0.74 Å). Moreover, these results are supported by the low angle shift of XRD peaks, which are correlated with the distortion of the ZnO crystal structure due to the difference of the ionic radius.

As shown in Table, the particle size of the Mg-doped ZnO nanoparticles were in the range of 26.88–21.6. The particle size (D) of the samples decreases with increasing Mg concentration ratio, while the dislocation density (δ) of the samples increases with Mg concentration. This was due to the inverse ratio between δ and values. The locality of the atoms and their displacements (u parameters) were calculated. It was seen that it was increasing with increasing Mg ratio. The Zn-O bond lengths (L parameters) were in the range of 1.9759–1.9780 Å.

Literature indicated that, reactivity of an element increases as the values of Pauling electro-negativity decrease [68]. Mg is more chemically reactive than Zn, since the Pauling electro-negativity of Mg (1.31) is less than that of Zn (1.65). Thus, Mg can easily be replaced by Zn in the nanoparticle structure. Besides, Mg doping level is expected to change the morphology of ZnO NPs depending on the synthesis method. Mg doped ZnO nanoparticles synthesized via the solution precipitation technique by Iqbal et al. [69] showed a dopant concentration dependent morphology. According to the literature, morphology of Zn_1−x_Mg_x_O NPs may change depending on the synthesis route and this may also cause a significant change on the particles surface area [70,71]. It has also been reported that surface area of NPs decreases with increasing particle size and this causes a decrease in the concentration of ROS generated from nanoparticles [72].

The morphology and element concentrations of Mg doped ZnO NPs were characterized by SEM and EDX, respectively.

**Figure 4:**
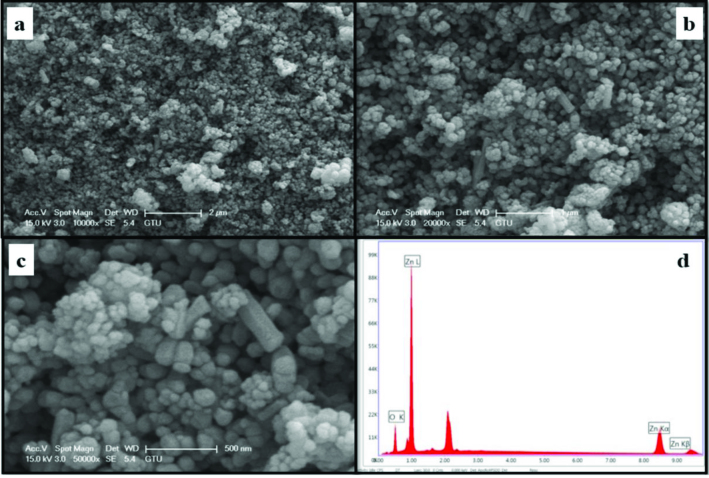
SEM micrographs of ZnO NPs with different magnifications, a. (×10,000), b. (×20,000), c. (×50,000), and d. energy-dispersive X-ray spectrum of ZnO NPs.

**Figure 5:**
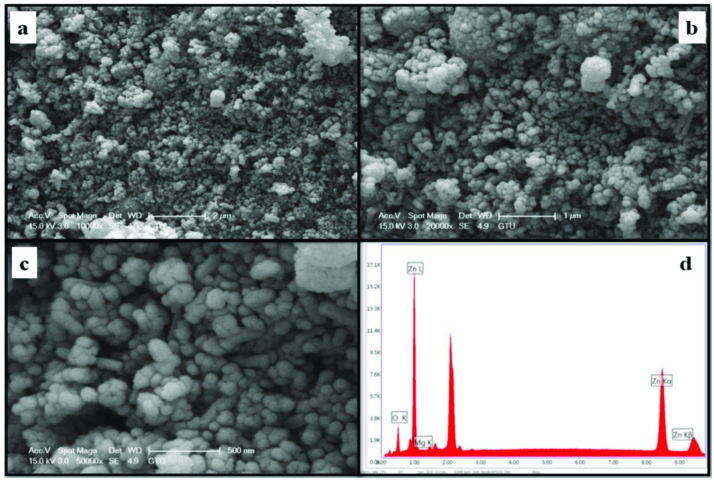
SEM micrographs of Zn_0.99_Mg_0.01_O NPs with different magnifications a. (×10,000), b. (×20,000), c. (×50,000), and d. energy-dispersive X-ray spectrum of Zn_0.99_Mg_0.01_O NPs.

**Figure 6:**
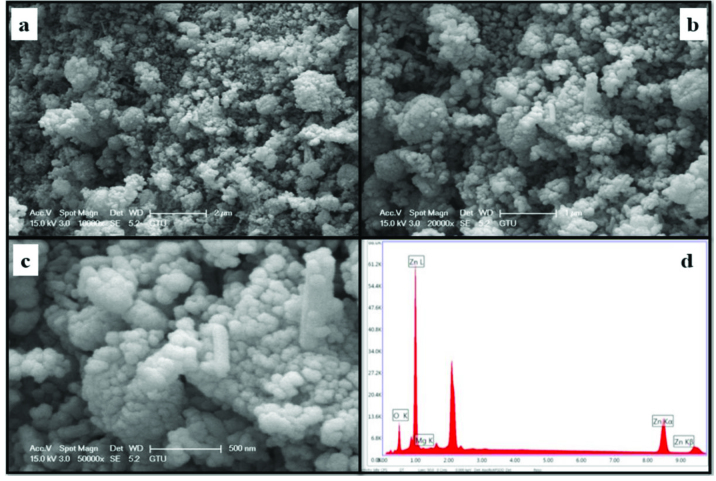
SEM micrographs of Zn_0.98_Mg_0.02_O NPs with different magnifications a. (×10,000), b. (×20,000), c. (×50,000), and d. energy-dispersive X-ray spectrum of Zn_0.98_Mg_0.02_O NPs.

**Figure 7:**
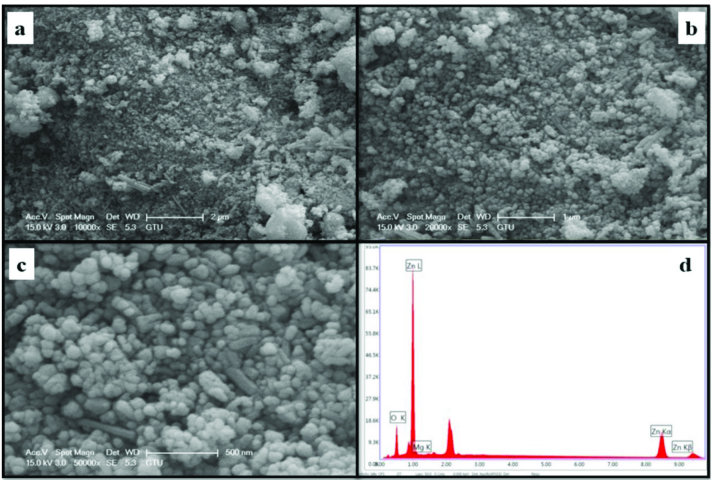
SEM micrographs of Zn_0.96_Mg_0.04_O NPs with different magnifications a. (×10,000), b. (×20,000), c. (×50,000), and d. energy-dispersive X-ray spectrum of Zn_0.96_Mg_0.04_O NPs.

**Figure 8:**
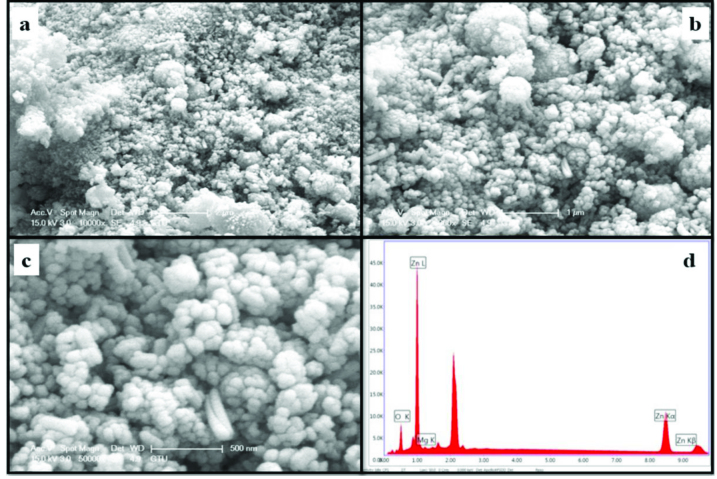
SEM micrographs of Zn_0.95_Mg_0.05_O NPs with different magnifications a. (×10,000), b. (×20,000), c. (×50,000), and d. energy-dispersive X-ray spectrum of Zn_0.95_Mg_0.05_O NPs.

**Figure 9:**
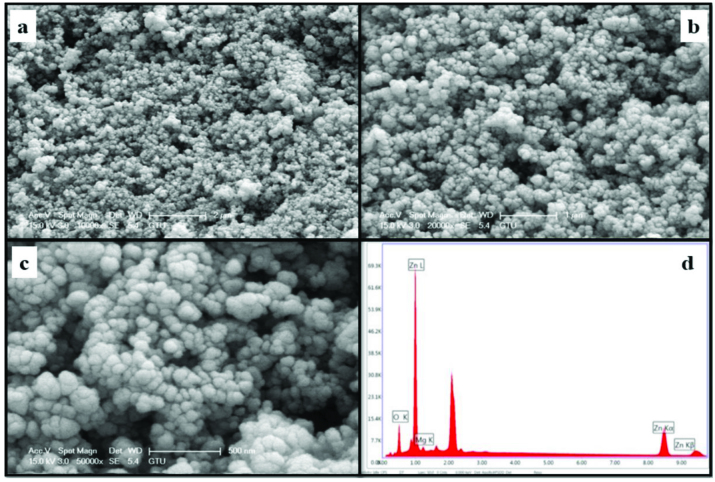
SEM micrographs of Zn_0.90_Mg_0.10_O NPs with different magnifications a. (×10,000), b. (×20,000), c. (×50,000), and d. energy-dispersive X-ray spectrum of Zn_0.90_Mg_0.10_O NPs.

**Figure 10:**
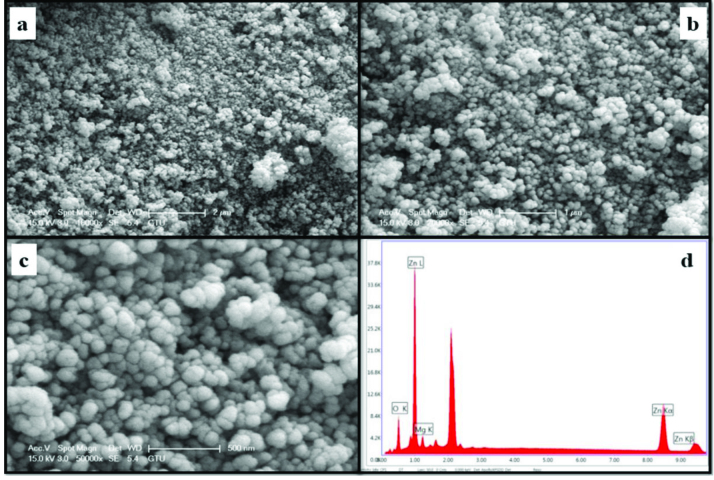
SEM micrographs of Zn_0.80_Mg_0.20_O NPs with different magnifications a. (×10,000), b. (×20,000), c. (×50,000), and d. energy-dispersive X-ray spectrum of Zn_0.80_Mg_0.20_O NPs.

**Figure 11:**
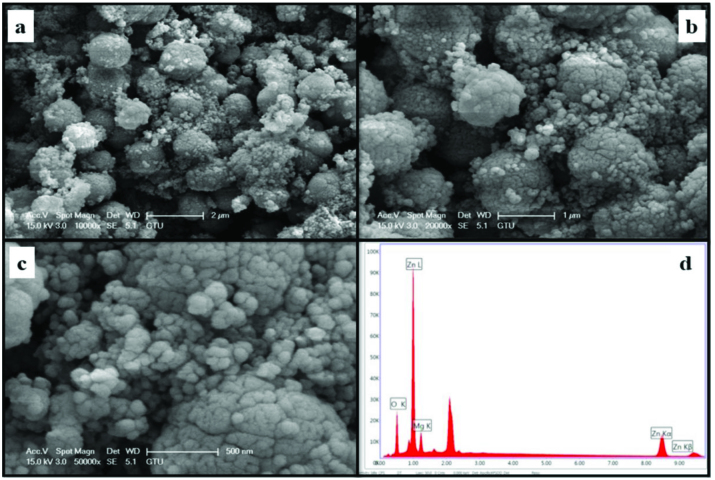
SEM micrographs of Zn_0.70_Mg_0.30_O NPs with different magnifications a. (×10,000), b. (×20,000), c. (×50,000), and d. energy-dispersive X-ray spectrum of Zn_0.70_Mg_0.30_O NPs.

Figures 4–11 show SEM micrographs of Zn_1−x_Mg_x_O NPs with varying Mg doping levels i.e. 0%, 1%, 2%, 4%, 5%, 10%, 20%, and 30%. The malting and agglomeration of Zn_1−x_Mg_x_O NPs increase when the concentration of Mg was increased as seen in the figures. From the SEM micrographs, Zn_1−x_Mg_x_O NPs were dense, quasi spherical and agglomerating. At the maximum concentration (x = 0.30), as shown in Figure 11, the highest agglomeration and a pellet like dense morphology were observed.

**Figure 12:**
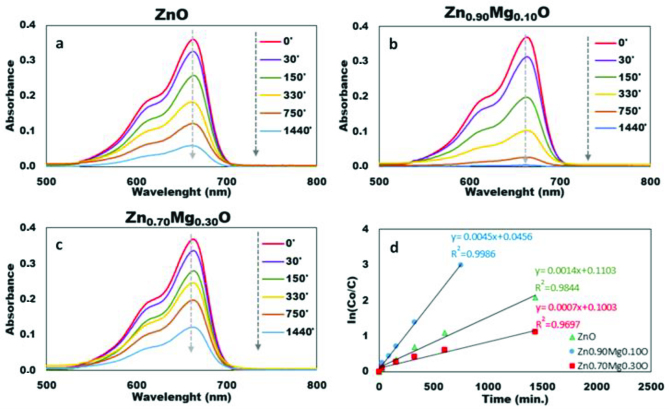
UV–vis spectra of MeB as a function of time in presence of a. ZnO, b. Zn0.90 Mg0.10 O, and c. Zn_0.70_Mg_0.30_O under ambient light irradiation, d. photo degradation kinetics of MeB.

Figures 12a–12c show the UV-visible absorption spectra of free (unadsorbed) MeB with the variation of time. Attenuation of the intensity of the absorption band at 664 nm indicated the degradation of MeB by bot ZnO and Zn1−x Mgx O photocatalysts. The characteristic band at 664 nm disappeared after 24 h under the effect of ambient light irradiation indicating the complete degradation of MeB by Zn0.90 Mg0.10 O nanoparticles. Another control experiment was conducted without nanoparticles under same experimental conditions. It was observed that, MeB was not degraded without ZnO or Mg-doped ZnO nanoparticles within 24 h.

The kinetics of MeB photo degradation shown in Figure 12d could be described by a first order kinetics in agreement with the Langmuir-Hinshelwood model:

r=-dCdt=k1k1C01+k1C0

where k_1_ is the adsorption coefficient, k_2_ is the specific rate constant for degradation and C_o_ is the initial concentration of MeB. The integrated form of the above equation can be written as follows:

t=1k1k2lnC0C+C0-Ck2

If the concentration (C) is very low, then the second term of the equation becomes very small compared to the first one and the equation can be simplified to:

lnC0=k1k2t≈k′t

The plot of ln(C_o_/C) versus irradiation time represents a straight line, whose slope is equal to the apparent first order rate constant (k’). Apparent photodegradation rate constants for undoped ZnO, Zn_0.90_Mg_0.10_O and Zn_0.70_Mg_0.30_O were 0.0014, 0.0045, and 0.0007 min^−1^, respectively. Results were consistent with the relevant literature related with MeB degradation [75,76].

Under the effect of electromagnetic radiation (light) the generation of electron-hole pairs between valence bands (VB) and conduction bands (CB) of ZnO NPs are predominantly responsible for the degradation of organic pollutants like MeB via ROS formation [77]. Decolorization of MeB is considered as a demonstrative evidence of its degradation.

ROS formation on the surface of zinc oxide nanoparticles starts with the excitation of electrons in the filled valence band (VB) with electromagnetic radiation having the energy higher than the value of zinc oxides band gap (~3.3 eV) [78]. Excited electrons are moved to an empty conduction band (CB) to form electron holes in the VB (h^+^_VB_) and free electrons in the CB (e^−^_CB_) (Eq. 1). h^+^_VB_ and e^−^_CB_ pairs migrate to the surface of the ZnO nanoparticles and they are involved in redox reactions resulting ROS formation in accordance with the below-mentioned mechanisms [78–81]. While h^+^_VB_ reacts with H_2_O molecules in aqueous ZnO suspensions to form OH* and H^+^, free electrons in the CB react with dissolved O_2_ molecules to transform them into superoxide anion radicals (O*_2_−) which in turn react with H^+^ to generate hydroperoxyl radicals (HO_2_) (Eqs.2–4).

To form hydrogen peroxide, hydroperoxyl radicals may collide with each other, or they can also interact with CB electrons and H^+^ ions (Eqs. 5–7). H_2_O_2_ will then react with O*_2_− to form OH* which is a powerful oxidizing agent (Eq. 8).ROS would be responsible from both antibacterial properties and degradation of organic molecules like MeB into less toxic degradation products such as CO_2_ and H_2_O (Eq. 9) [53].

(1)ZnO→hvZnO+hvB++eCB-

(2)hVB++H2O→H++OH*

(3)eCB-+O2→O2*-

(4)O2*-+H+→HO2*

(5)HO2*+HO2*→H2O2+O2

(6)HO2*+e-→HO2*-

(7)HO2*-+H+→H2O2

(8)H2O2+O2*-→OH*+O2

(9)MeB+(O2,O2*-,HOO*orHO*)→CO2+H2O

**Figure 13:**
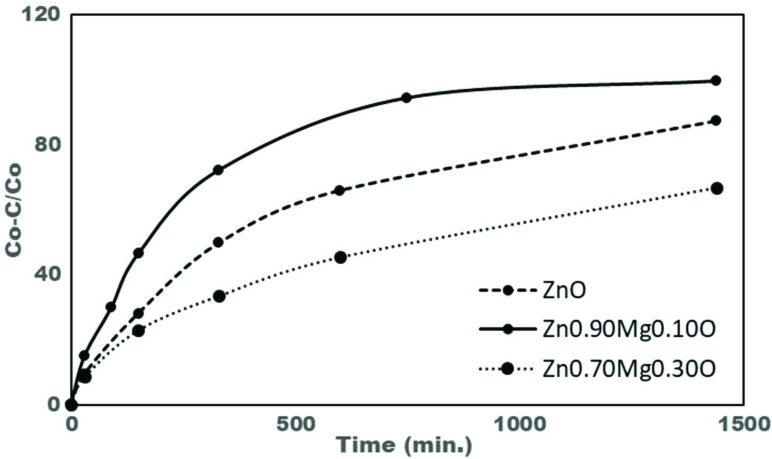
Decolorization efficiencies of ZnO, Zn_0.90_Mg_0.10_O and Zn_0.70_Mg_0.30_O as a function of time.

Figure 13 shows the decolorization efficiencies of ZnO, Zn_0.90_Mg_0.10_O and Zn_0.70_Mg_0.30_O as a function of time. ZnO samples doped by 10% Mg exhibited much higher photocatalytic activity than that of pure ZnO and Zn_0.70_Mg_0.30_O nanoparticles. At the end of 1440 min, decolorization efficiency of Zn_0.90_Mg_0.10_O was around 99.7% whereas the relevant values for ZnO and Zn_0.70_Mg_0.30_O was 87.4% and 66.8% respectively.

### 3.3. Antibacterial properties

Antibacterial activity of ZnO NPs synthesized by the hydrothermal and the vapor deposition methods is well known in literature [82–85]. Nevertheless, concentration dependent antibacterial activity of ZnO NPs synthesized by the sol-gel method still have unsolved issues.

In this study, activity of Zn_1−x_Mg_x_O NPs against bacteria was examined with different routes. To determine the antibacterial activity of NPs, the Kirby–Bauer disc diffusion method and the agar well diffusion method was first applied. In disc diffusion method, powder form of nanoparticles was pelleted using a hydraulic press, then they were transferred carefully on to the agar plates inoculated with *E. coli.* Similar to the procedure used in the Kirby–Bauer disc diffusion method, the agar plate surfaces were inoculated with the 1×10^6^ cfu/mL *E. coli* suspension. Then, a hole for each test sample was punched aseptically with a sterile cork borer, and NPs were placed into wells with three different concentrations in two ways; as solid powder form and as colloidal nanoparticle suspensions in water (30 μL each). Then, agar plates were incubated at 37 ⇄C for overnight. In both tests, the zone of inhibition could not be formed. As a third route, standard agar plate count method was used.

**Figure 14:**
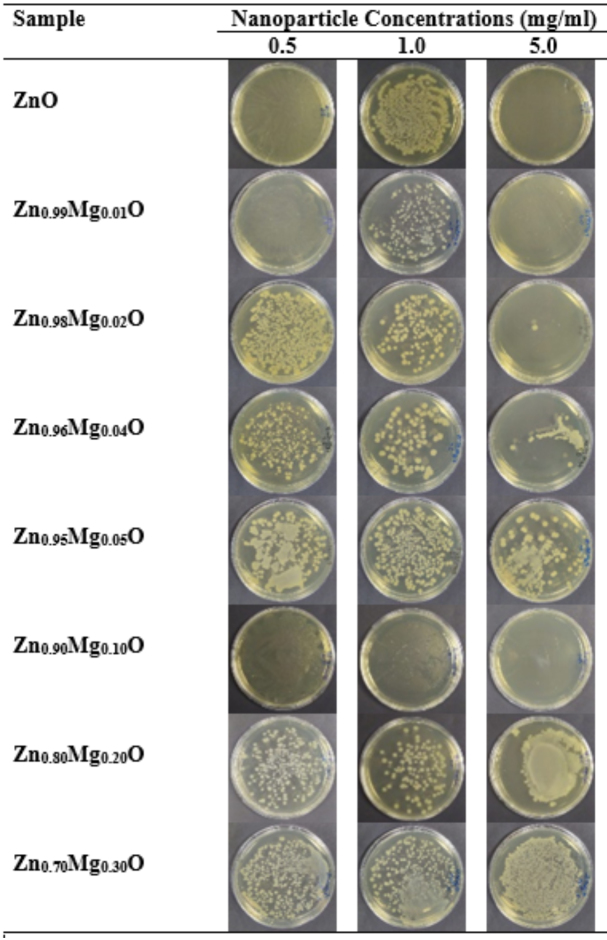
Photographs of agar plate counting method for ZnO and Zn_1−x_Mg_x_O NPs by plate counting. *E. coli* suspensions were treated with different concentrations (0.5, 1.0 and 5.0 mg/mL) of NPs in test tubes for overnight in liquid phase and a small amount of *E. coli* suspension (30μL) treated with NPs from each test tube was inoculated into agar plates. After incubation period, number of colonies in agar plates were observed.

Antibacterial activities of ZnO and Zn_1−x_Mg_x_O nanoparticles were studied against gram-negative *Escherichia coli* K12 strains. In our study, nanoparticle concentrations were determined as 0.5 mg/mL, 1.0 mg/mL, and 5.0 mg/mL (corresponding to 6, 12, and 60 mM for ZnO) which was almost 5 times higher than the range reported in the literature [40,85,86]. For undoped ZnO and in low Mg doping levels, antibacterial activity of NPs exhibited both positive and negative concentration dependent behavior (Figure 14). After an overnight incubation period, ZnO NPs showed an antibacterial activity in 0.5 mg/mL and 5.0 mg/mL concentrations. However, at the concentration of 1.0 mg/mL any antibacterial activity was not observed. The same results were obtained for the Zn_0.99_Mg_0.01_O sample with lower colony numbers at the same concentration (1.0 mg/mL). In this study, the lowest nanoparticle concentration was 0.5 mg/mL. For ZnO and Zn_0.9_Mg_0.01_O, this lowest concentration may not trigger the defense mechanism of bacteria. As nanoparticle concentration increases antibacterial activity that was observed at low NP concentration disappeared at 1.0 mg/mL. It may be due to some upregulations in bacterial genome as it was stated in literature [87]. Nevertheless, at high ROS concentration due to increased NP concentration (5.0 mg/mL) bacteria could not survived. In a previous study conducted by Khan et al. [88], the effect of type and concentration of ROS on leading to cell death was investigated. Their results proved the varying toxic effects of singlet oxygen, hydroxyl radicals and hydroxyl ions at different concentrations. In another study, effect of Zn_0.15_Mg_0.85_O NPs on Bacillus subtilis proteome was investigated by controlling the protein profiles by one-dimensional sodium dodecyl sulfate polyacrylamide gel electrophoresis (SDS-PAGE). It was mentioned that, after 5.5 h of exposure to 0.05 mg/mL Zn_0.15_Mg_0.85_O NPs, among more than 1550 membrane proteins resolved, the most upregulated ones are related with the toxic metal export (CzcD and CadA), redox conditions and defense (AhpC, BrxA, and BrxB) against ROS [87]. Researchers indicated that bacteria can modify its proteomic structure when they are exposed to harsh environmental conditions.

Mg is an essential element for both prokaryotic and eukaryotic organisms. From Zn_0.98_Mg_0.02_O to Zn_0.70_Mg_0.30_O (except Zn_0.90_Mg_0.10_O) due to increasing Mg content, antibacterial activity of nanoparticles weakened at both high and low concentrations (Figure 14).

The reenhancement in the antibacterial activity of Zn_0.90_Mg_0.10_O may not be due to the variation in particle size or surface morphology according to SEM-EDX analysis, since our SEM results showed no change in the morphology of nanoparticles. ICP-OES results also proved that ZnO and Mg doped ZnO nanoparticles were quite stable. Presence of Zn^2+^ was not detected in any sample for either pure water or PBS. An insignificant range of 0.145–0.253 μg/L (ppb) was found for Mg^2+^ concentration among all the concentrations of samples. Mg^2+^ levels were very close for all samples, regardless of nanoparticle amount or Mg doping level.

Result indicates that zinc and magnesium in the nanoparticles were not leached in any way and nanoparticles do not dissolve in both water and PBS. Results consistent with XRD analysis (Figure 2) also proved that the nanoparticle structure was not destroyed in pure water and PBS. XRD results indicated a detectable peak corresponded to MgO phase in Zn_0.70_Mg_0.30_O sample.

### 3.4. Blood compatibilities

Nano sized materials may change or deform the morphology of red blood cells (RBCs or erythrocytes) and cause hemolysis, i.e. lysis of cell membrane when they interact with erythrocytes while, ZnO nano particles are regarded as non-toxic, biosafe and possibly biocompatible [28,78]. NPs including ZnO are usually administered intravenously in drug delivery and medical imaging applications [89,90]. However, catalytic activity of the nanoparticle surfaces may cause ROS generation and resulting an increase in oxidative stress leading to cellular damage [53,72,61].

Determination of the hemolytic potential of nanoparticles on human erythrocytes is an alternative method for in vivo testing of biological properties. In a literature study [78], human RBCs were found to be very stable after treatment with 0.100 mg/mL of ZnO NPs (~25 nm in diameter) synthesized by homogeneous gas phase condensation method. In another study, ZnO nanoparticles (~30 nm in diameter) synthesized by solution precipitation method showed no apparent hemolysis up to 0.600 mg/mL [41].

**Figure 15:**
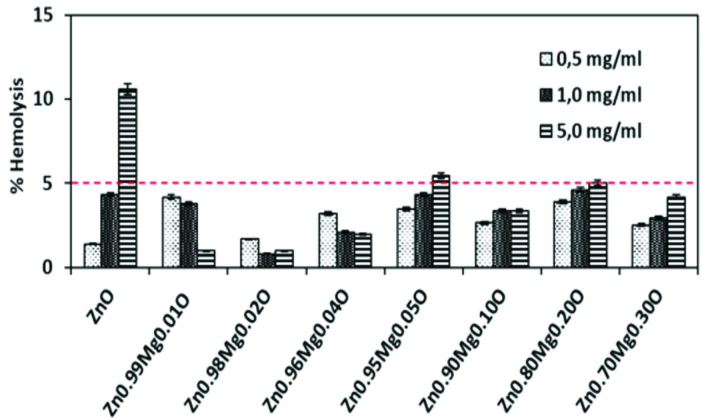
Hemolysis ratios of human erythrocytes after incubation with ZnO and Zn_1−x_Mg_x_O nanoparticles.

In this study, human erythrocytes drawn from healthy volunteers were used to investigate the hemolytic potentials of ZnO and Zn_1−x_Mg_x_O nanoparticles. Figure 15 shows the hemolysis percentages of blood samples in contact with ZnO and Zn_1−x_Mg_x_O nanoparticles. Results indicated that hemolysis ratio and nanoparticle concentration were directly proportional in case of ZnO and lower than the acceptable level of 5% [91] up to 5.0 mg/mL. In this study, nanoparticle concentrations were 5 times higher compared to the relevant literature [41,85,86].

In the study of Iqbal et al. [92], biocompatibility of cobalt doped ZnO NPs synthesized by coprecipitation method were investigated. They found concentration dependent increments in hemolysis percentages and relatively low hemolysis ratios up to 0.25 mg/mL nanoparticle concentration.In the present study, low hemolytic activity ratios of ZnO and Zn_1−x_Mg_x_O nanoparticles attributed to the homogeneous and highly pure structure of nanoparticles proved by SEM-EDX (Figures 4–11). Also, all Mg doped ZnO nanoparticles (except Zn0.90 Mg0.10 O at 5.0 mg/mL) showed lower hemolytic activities and hemolysis ratios were concentration dependent for most of the nanoparticles. All Zn_1−x_Mg_x_O nanoparticles indicated much lower hemolytic activities than that of pure ZnO nanoparticles at 5.0 mg/mL. Especially for Zn_0.90_Mg_0.10_O sample, both higher antibacterial properties and lower hemolytic ratios were obtained.

## 4. Conclusion

Pure, chemically and morphologically homogenous, antibacterial and hemocompatible Zn_1−x_Mg_x_O (x = 0.0, 0.01, 0.02, 0.04, 0.05, 0.10, 0.20, and 0.30) nanostructures were successfully synthesized by sol-gel method. Phase composition, antibacterial properties and hemolytic potential of the nanostructured powders were characterized by XRD, SEM-EDX, agar plate counting method and blood compatibility (hemolysis) tests respectively. Results from SEM micrographs demonstrated that Zn_1−x_Mg_x_O NPs were dense, quasi spherical, and agglomerated. An increment was observed in malting and agglomeration of Zn_1−x_Mg_x_ O NPs in increased Mg dopant concentration. Additionally, two different phases of ZnO and MgO were obtained, when the Mg concentration was higher than 20%. Zn0.90 Mg0.10 O and Zn0.70 Mg0.30 O nanoparticles exhibited better photocatalytic effect on MeB than that of pure ZnO under ambient visible light irradiation. The results of photocatalytic activity tests showed that Zn_0.90_Mg_0.10_O exhibited the highest activity, whereas Zn_0.70_Mg_0.30_O showed the lowest. The results of blood compatibility tests showed acceptable (<5%) levels [91] of hemolysis ratios for most of our nanoparticle applications. In case of ZnO, hemolysis ratios were below the acceptable level up to 5.0 mg/ml and nanoparticle concentration was also directly proportional to the hemolysis ratios. Additionally, in this study, antibacterial activity of NPs wasobtained in both 0.5 mg/mL and 5.0 mg/mL concentrations for ZnO, Zn_0.99_Mg_0.01_O and Zn_0.90_Mg_0.10_O against *E. coli* . Among these, Zn_0.90_Mg_0.10_O showed both higher antibacterial properties and lower hemolytic ratios. As known, in addition to low and/or acceptable hemolytic activity, broad spectrum, high stability, minimum drug resistance and side effects are desired features for antimicrobial drugs. In this respect, Mg doped ZnO nanoparticles and especially Zn_0.90_Mg_0.10_O could have commercial applications in UV sterilization systems [93].The studies and analyses in this study should give valuable ideas to design and develop new antibacterial nanosized materials for pharmaceutical industry that looking for alternative antibacterial agents in today’s world while the effects of classical antibiotics are continuously decreased in recent years.
